# Development and Applications in Intelligent Sports of Hydrogel-Based Triboelectric Nanogenerators

**DOI:** 10.3390/ma18010033

**Published:** 2024-12-25

**Authors:** Guishan Feng, Yunlu Wang, Dongsheng Liu, Zihang Cheng, Qingyang Feng, Hongwei Wang, Wei Han, Changjun Jia

**Affiliations:** 1 School of Competitive Sports, Shandong Sport University, Rizhao 276826, China; fengguishan@sdpei.edu.cn (G.F.); wanghongwei@sdpei.edu.cn (H.W.); 2 Physical Education Department, Northeastern University, Shenyang 110819, China; 2371501@stu.neu.edu.cn (Y.W.); 2371489@stu.neu.edu.cn (D.L.); 2301409@stu.neu.edu.cn (Z.C.); 2301410@stu.neu.edu.cn (Q.F.); 3 Department of Public Teaching, Qilu Medical University, Zibo 255300, China

**Keywords:** hydrogel-based, TENG, intelligent sports, development and applications

## Abstract

As an emerging self-powered technology, triboelectric nanogenerators have the characteristics of a simple structure, high conversion efficiency, diverse material selection, and stable output. Hydrogels have the advantages of flexibility, extensibility, and shape adaptability, which means that hydrogel-based triboelectric nanogenerators (H-TENGs) have high flexibility, self-healing abilities, conductivity, and fatigue resistance. They can still operate normally in scenarios involving bending, pressing, stretching, and folding. H-TENGs offer a method of versatile and sustainable innovation in sports monitoring. This review elucidates the working principles and modes of H-TENGs, examines H-TENG characteristics that are relevant to intelligent sports, and summarizes their applications in this field. This paper concludes with a discussion on the development and applications of H-TENGs in intelligent sports.

## 1. Introduction

The incorporation of advanced intelligent sports technologies, including sensing technology, big data analysis, artificial intelligence, and virtual reality, into sporting environments and services has enhanced their safety, scientific basis, efficiency, and convenience. The widespread application of wearable devices and sensors has brought the sports field into the digital age. Various sensing technologies in intelligent sports offer benefits like high sensitivity and multifunctionality [1,2]. However, traditional wearable devices often have poor comfort and a high cost, and they also have the common disadvantage of requiring an external power source for their operation [3,4,5,6]. Developing a sustainable, maintenance-free sensor for monitoring human motion data is essential.

In 2012, the academician Zhonglin Wang introduced a triboelectric nanogenerator (TENG) characterized by its cost-effectiveness, simplicity, high efficiency, and versatile material options, enabling the conversion of distributed and irregular mechanical energy from human motion into electrical energy [7,8,9,10,11]. The TENG functions as a self-powered sensor for tactile pressure, acceleration, and motion detection, eliminating the need for an external power source [12,13,14]. Through their integration with signal processing and transmission modules, enhanced triboelectric sensing signals can be produced, and wireless sensing can be further achieved in TENG-based self-powered systems. Flexible wearable sensors offer advantages like portability, flexibility, compliance, and cost-effectiveness, demonstrating significant potential for real-time, continuous monitoring of physiological and biological states [15,16,17,18,19,20,21,22,23]. To address the discomfort caused by high-stiffness materials, researchers are investigating conductive materials like carbon nanotubes [24], graphene [25], metal particles [26], and conductive polymers [27]. These have been effectively integrated with elastomeric composites to create flexible sensors with superior electrical and mechanical properties [28]. In practical applications, the performance of these devices is still significantly impacted by their insufficient sensitivity, poor biocompatibility, and limited detection range, as well as delamination between the electronic conductors and substrates [29,30].

Hydrogels have a unique three-dimensional elastic hydrated polymer network structure, which can hold and stabilize a high proportion of water. Hydrogels’ mechanical properties, including their toughness, extensibility, and fluidity, can be flexibly modified during synthesis [31]. Hydrogels also have excellent transparency, excellent extensibility, good biocompatibility, and little impact on the environment [32,33,34,35]. In particular, the conductivity exhibited by hydrogels not only enables their resistance and charge carrier density to be fine-tuned and optimized, but it also allows developers to select chemical ion types according to their needs. These characteristics promote efficient integration between biological and electronic systems, demonstrating unprecedented flexibility and adaptability [36,37]. Equally, hydrogels can have self-healing, self-adhesive, or frost-resistant properties [38,39,40,41,42,43]. Researchers can flexibly make different types of hydrogels with different materials and methods according to their requirements, as shown in Table 1. Based on the above characteristics, hydrogels are the ideal material for TENGs. As shown in Table 2, an H-TENG can overcome the need for an external power supply in optical, resistive, ultrasonic, and MEMS sensors, among others. Compared with other new self-powered sensors, such as piezoelectric, thermoelectric, and photoelectric sensors, hydrogel-based triboelectric nanogenerators (H-TENGs) have shown unique advantages in their conformability, manufacturing cost, extendibility, environmental adaptability, and low-frequency micromotion response, especially in sports scenarios that require high-frequency monitoring, demonstrating enormous application potential.

**Figure 1:**
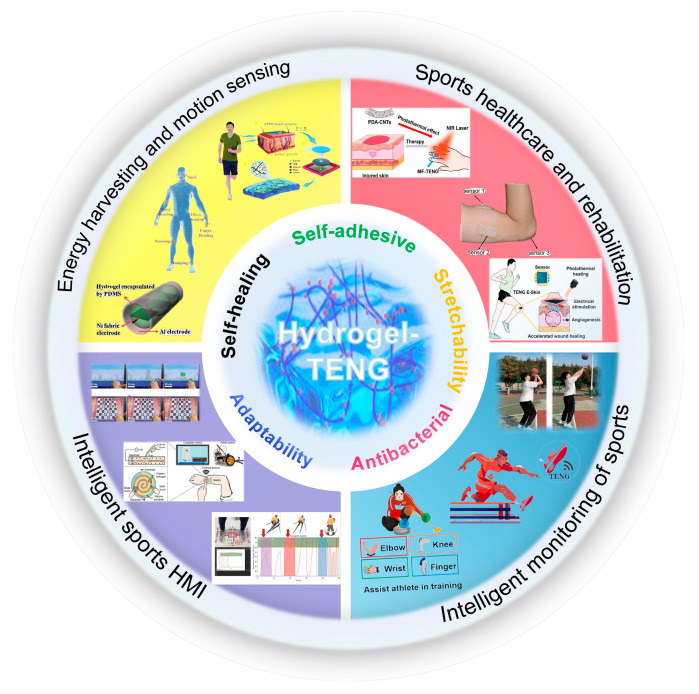
Illustration of hydrogel-based triboelectric nanogenerators used for intelligent sports applications. Images for ‘Energy harvesting and motion sensing’ were reproduced with permission from Ref. [48] (copyright 2016, John Wiley and Sons), Ref. [49] (copyright 2023, Elsevier), and Ref. [50] (copyright 2022, John Wiley and Sons). Images for ‘Sports healthcare and rehabilitation’ were reproduced with permission from Refs. [51,52] (copyright 2021, American Chemical Society) and Ref. [53] (copyright 2022, Elsevier). Images for ‘Intelligent monitoring of sports’ were reproduced with permission from Ref. [54] (copyright 2023, AlP Publishing) and Refs. [55,56] (copyright 2024, Elsevier). Images for ‘Intelligent sports HMI’ were reproduced with permission from Ref. [57] (copyright 2022, John Wiley and Sons), Ref. [58] (copyright 2022, Elsevier), and Ref. [59] (copyright 2024, John Wiley and Sons).

This review introduces the primary operating principles of H-TENGs, focusing on the vertical contact separation and single-electrode working mode. We also analyzed certain characteristics of H-TENGs suitable for intelligent sports, such as self-adhesiveness, stretchability, antibacterial properties, cold and heat resistance, and self-healing properties. The latest research advancements in H-TENGs for intelligent sports are examined across four areas: energy harvesting and motion sensing during daily exercise, human sports healthcare and rehabilitation, intelligent monitoring in sports, and human–machine interfaces in intelligent sports. Figure 1 illustrates the use of H-TENGs in intelligent sports. H-TENGs are anticipated to advance in four areas—hydrogel material development, multifunctional sensor systems, market application expansion, and human–computer interface optimization—with the goal of enhancing their practical efficiency.

## 2. Operating Principle of H-TENGs

Human motion is complex and irregular. The nervous system activates various periodic and irregular limb movements (such as swinging arms, stepping, and even breathing) through muscles, bones, and joints. These movements produce distributed, irregular, and low-frequency mechanical energy, which can be converted into electrical energy by TENG devices [60]. TENG devices are manufactured easily and affordably, offering significant potential for application in self-powered systems for monitoring, medical, and electronic products [61].

The selection of electrode materials enables TENGs to have better performance in terms of energy conversion rate and durability, with reduced manufacturing costs and greater environmental adaptability [62]. Electrode materials often comprise high-conductivity or cost-effective metals like copper, nickel, aluminum, or zinc [63]. The advantages of high conductivity, durability, and simple manufacturing make hydrogels an ideal choice for TENG electrode materials. The fundamental modes of TENGs are categorized into five types: vertical contact–separation (C-S) mode, lateral sliding mode, single-electrode mode, independent frictional layer mode [64], and rolling mode [65]. The working principle of H-TENGs is similar to that of general TENGs. As different materials come into contact and move relative to each other, the mechanical energy in human motion is converted into electrical energy through the coupling of contact electrification and the electrostatic induction effect. The main operating modes of H-TENGs are the vertical C-S mode and the single-electrode mode. The two working modes are introduced in the following sections.

### 2.1. Vertical Contact–Separation Mode

The vertical C-S mode of TENGs operates through the contact and separation motion of triboelectric layers with opposite charges. Hydrogels are generally flexible and stretchable electrode materials for H-TENGs. When two triboelectric layers with different polarities are in contact with external forces, electrons will be transferred from the positive triboelectric material to the negative triboelectric material due to the contact electrification effect. A potential difference will be generated when the two triboelectric layers are separated, causing the charge on the triboelectric layers to rearrange the anions and cations in the hydrogel electrode. The anions in the hydrogel will move to the positive triboelectric layer, while the cations will be moved to the other side due to electrostatic induction. Meanwhile, the negative triboelectric layer will experience the opposite. If the hydrogel electrodes are connected by an external circuit, the potential difference will induce an electron flow to eliminate the difference. An alternating current can be generated by repeatedly connecting and separating the positive and negative triboelectric layers periodically [66,67,68].

**Figure 2:**
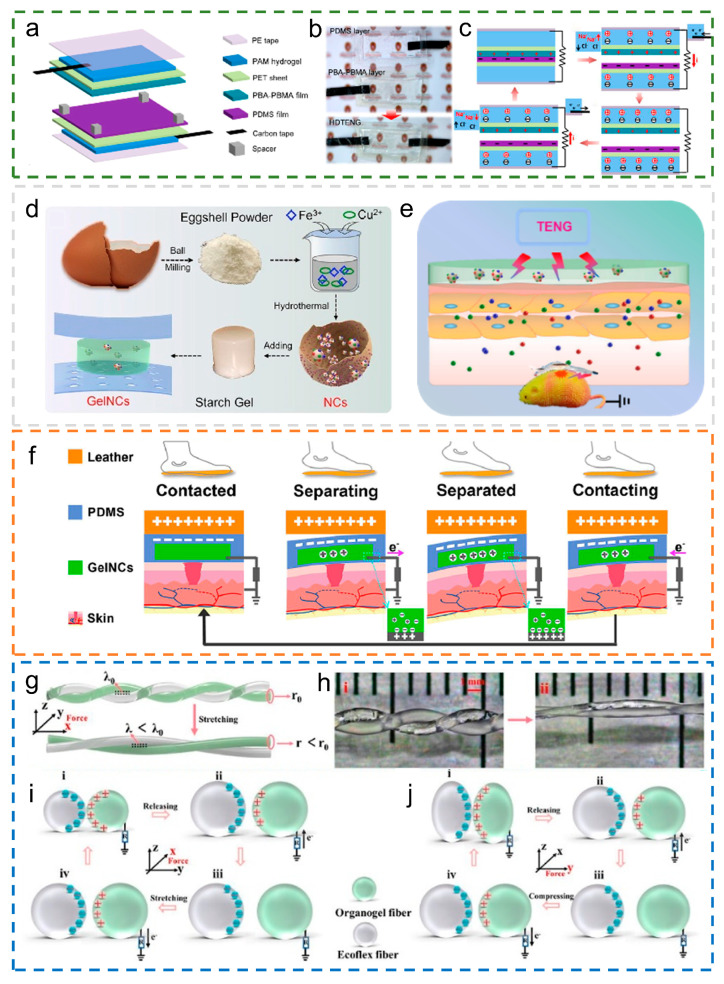
The working mechanism of a hydrogel-based TENG. (**a**–**c**) Illustration of the HD-TENG, featuring the two-electrode setup and the vertical contact–separation operational mode. This content has been reproduced with permission from Ref. [69] (copyright 2020, American Chemical Society). (**d**–**f**) Schematic diagram of GelNC, a working model of the patch, and different walking stages. This content has been reproduced with permission from Ref. [70] (copyright 2024, Elsevier). (**g**) Schematic illustrating the two states of the double-helix fiber structure under stretching. (**h**) (**i**) is the microscopic images of OFS-TENG, and (**ii**) is the physical drawing of OFS-TENG that is stretched. (**i**,**j**) The mechanism of single-electrode operation in tensile and compressive modes of OFS-TENG, (**i**) represents contacted, (**ii**) represents separating, (**iii**) represents separated, (**iv**) represents contacting. This content has been reproduced with permission from Ref. [71] (copyright 2022, American Chemical Society).

Using the copolymer of poly (n-butyl acrylate-co-n-butyl methacrylate) (PBA-PBMA) as the tribopositive layer, polydimethylsiloxane (PDMS) as the tribonegative layer, and a polyacrylamide (PAM) hydrogel containing sodium chloride as the electrode, Mi et al. [69] developed a hydrogel-based dual-electrode triboelectric nanogenerator (HD-TENG). Figure 2a illustrates the HD-TENG structure operating in contact–separation mode, utilizing PET film to support the triboelectric layers. Figure 2b illustrates the PDMS and PBA-PBMA layers, alongside a physical image of the HD-TENG, highlighting its high transparency. Figure 2c illustrates the HD-TENG mechanism, where contact between the two layers induces charge and electron transfer due to the triboelectric polarity difference between PDMS and PBA-PBMA. The separation of the two layers induces a potential difference, leading to Na^+^ and Cl^−^ ion redistribution in the PAM ionic hydrogel electrodes on the triboelectric materials. Connecting the two PAM hydrogel electrodes generates a potential difference, causing electron flow through the external circuit. The energy produced by HD-TENGs, once converted to direct current via a rectification bridge, can power electronic devices such as pedometers, digital timers, and swimming watches.

### 2.2. Single-Electrode Mode

Unlike the dual-electrode TENG operating in the vertical C-S mode, the single-electrode mode only uses one electrode [72]. In the single-electrode mode of TENGs, the triboelectric layer typically comprises a continuous structure, which includes a triboelectric region and a collection region (grounding). Upon contact with an external object, the surfaces involved generate triboelectric charges of opposite polarity due to differences in their positions within the triboelectric series. When the external objects are separated from the triboelectric layer, these triboelectric charges remain uncompensated, leading to potential differences. Consequently, due to the electrostatic induction effect, positive (or negative) charges are induced in the collection area (hydrogel electrode), prompting the flow of free electrons from the collection area to the ground (or vice versa). Conversely, when the external objects approach the triboelectric layer, free electrons flow from the ground to the collection area (or vice versa), resulting in voltage and current signals of opposite polarity. An alternating current can be generated by periodically and repeatedly connecting and separating the triboelectric layers. The single-electrode-mode TENG features a straightforward design and operation, minimizing material and circuit complexity [73,74].

To promote wound healing and realize real-time monitoring of human gait, Zhang et al. [70] prepared a new eggshell@CuFe_2_O_4_ nanocomposite, which has a unique structure and inherent antibacterial properties. Figure 2d illustrates the typical preparation method of GelNC. Sandwiching GelNC between two layers of PDMS film forms the iTENG patch. Figure 2e illustrates the use of GelNC-based patches on a wound and depicts the working principle of the iTENG for promoting wound healing. The working mechanism of human gait monitoring using the iTENG is shown in Figure 2f.

With the aim of unpowered and suturable strain monitoring of implanted ligaments, Sheng et al. [71] introduced a helical sensor utilizing organogel/silicone fibers, functioning as a triboelectric nanogenerator (OFS-TENG). The single-electrode-mode OFS-TENG features a helical core made of organic gel fibers, which act as conductive electrodes, and silicone fibers, which function as dielectric layers. Figure 2g,h depict the interaction mechanism of the OFS-TENG during both stretching and compression. Figure 2i,j explore in detail the charge generation mechanism under two working modes. The OFS-TENG features a double-helix structure of intertwined organic gel and silicone fibers, providing enhanced stability and exceptional stretchability.

## 3. Characterization of H-TENGs for Intelligent Sports Applications

Among the diverse array of flexible electrode materials, hydrogels are particularly noteworthy due to their self-adhesive properties, remarkable stretchability, antimicrobial efficacy, excellent compatibility with both cold and hot temperatures, and self-healing capabilities. Hydrogels have been extensively utilized in the monitoring of human motion and health. Their advanced functional characteristics not only open up numerous possibilities in the realm of smart motion monitoring but also highlight their substantial potential in this field.

**Figure 3:**
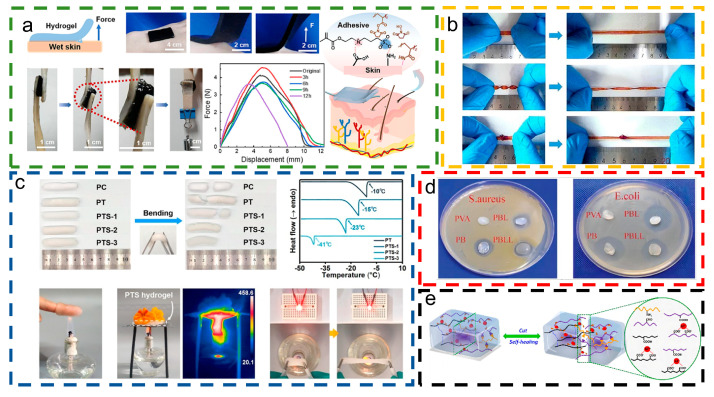
Characterization of H-TENGs for intelligent sports applications. (**a**) Schematic of adhesion performance testing of PAS/CNF/MXene hydrogels and adhesion mechanism. This content has been reproduced with permission from Ref. [75] (copyright 2024, Elsevier). (**b**) Stretched, twisted, and knotted hydrogel. This content has been reproduced with permission from Ref. [76] (copyright 2024, Royal Society of Chemistry). (**c**) Cold and fire resistance of PTS hydrogel. This content has been reproduced with permission from Ref. [77] (copyright 2024, Elsevier). (**d**) Bacterial inhibition of hydrogel. This content has been reproduced with permission from Ref. [59] (copyright 2024, John Wiley and Sons). (**e**) The self-healing mechanism of PAOAM-PDO hydrogel. This content has been reproduced with permission from Ref. [78] (copyright 2023, American Chemical Society).

In human motion monitoring, adhesion properties are vital for securing the sensor device to the skin, ensuring accurate and reliable data collection. Zhang et al. [75] engineered hydrogel strain sensors with outstanding conformal adhesion by incorporating cellulose nanofibers (CNFs) into a copolymer matrix of acrylic acid (AA), sulfobetaine methacrylate (SBMA), and N-hydroxysuccinimide (AA-NHS). Figure 3a evaluates the adhesion properties of the PAS/CNF/MXene hydrogel on wet skin, highlighting its exceptional adhesive performance. Applying a peeling force to the hydrogel’s end successfully elevates the skin without causing detachment. The integration of self-adhesive properties in hydrogel-based self-powered sensors has facilitated innovative applications in intelligent sports, allowing them to securely adhere to the human body.

In wearable sensing and monitoring applications, joints frequently serve as sites for sensor placement, making flexibility and stretchability crucial in material selection. Another important characteristic of hydrogels is their ability to stretch. Certain hydrogels can expand beyond their original length and grow to several times their size due to swelling of the polymer matrix and interactions with water. As shown in Figure 3b, the MAGP hydrogel can be easily elongated to five times its initial length without fracturing, showcasing its impressive elongation capabilities. Di et al. [76] developed a dynamically crosslinked hydrogel that is ultra-tough, highly sensitive, and resistant to swelling. This type of hydrogel can endure various forms of deformation, including stretching, twisting, and knotting.

Hydrogels are extensively acknowledged as flexible electrodes in both scientific research and practical applications; however, their substantial moisture content poses several challenges. Specifically, water within hydrogels is prone to freezing at low temperatures and evaporating at elevated temperatures. These characteristics significantly limit the use of hydrogels in extreme temperature conditions, thereby affecting their stability and reliability for long-term applications. Consequently, improving the temperature adaptability of hydrogels has emerged as a critical concern essential for the advancement and application of high-temperature triboelectric nanogenerator (H-TENG) technology. Han et al. [77] successfully synthesized a self-repairing, frost-resistant, and fire-retardant hydrogel strain sensor using eco-friendly natural biomaterials. As illustrated in Figure 3c, the PTS hydrogel can endure bending beyond 90° at −20 °C without breaking. It is also capable of withstanding brief exposure to vinyl flames, thereby protecting the skin from heat and burning sensations. The unique features of PTS hydrogel make it a promising candidate for intelligent sports monitoring and electronic device interaction applications.

The application of motion monitoring devices may facilitate bacterial proliferation, thereby increasing the risk of adverse reactions at the site of contact, such as skin infections or allergic responses, due to direct dermal contact. To mitigate this risk effectively, it is imperative to engineer hydrogels with strong antimicrobial properties. These properties are essential for safeguarding skin health and improving the precision and reliability of the monitoring data. Liu et al. [59] introduced an ionic co-hybridized antimicrobial hydrogel known as PBLL. As demonstrated in Figure 3d, the PBLL hydrogel exhibits inhibitory effects against *E. coli* and *S. aureus*. The unique mechanical characteristics of this hydrogel facilitate flexible adaptation to the movements of the body while minimizing the complications associated with secondary dressing fixation. This attribute is particularly valuable in biomedical settings and expands the possibilities for applications within the realm of intelligent sports.

Under dynamic and complex application environments (such as repeated pressure and high-strength stretching), performance degradation or even failure of hydrogels is inevitable. To satisfy the requirements of human motion monitoring, Zhao et al. [78] developed an organic hydrogel (PAOAM-PDO) with self-healing functions, among others. The self-healing mechanism of PAOAM-PDO is shown in Figure 3e. After the PAOAM-PDO was cut off due to dynamic and reversible metal coordination and Schiff base bonds, the two incisions of PAOAM-PDO were put into contact with each other for a while, and the recombination of PAOAM-PDO was realized. The self-healing ability of PAOAM-PDO is beneficial to maintaining the structural integrity and application reliability of H-TENGs.

The excellent adhesion of hydrogels makes them widely used in various fields of basic medicine, including drug delivery, wound healing, biomedical devices, and tissue engineering [79,80,81]. Owing to their exceptional mechanical attributes, self-healing hydrogels are increasingly favored for a range of electrical and biomedical applications. These materials demonstrate notable self-healing capabilities through diverse mechanisms, including metal–ligand and hydrogen bonding, reversible ionic crosslinking, dynamic covalent bonding, and supramolecular interactions. This self-healing capacity not only preserves the stability and reliability of the material’s properties but also substantially extends its lifespan [82,83,84].

## 4. Application of H-TENGs in Intelligent Sports

Due to their unique properties, hydrogel materials are highly suitable for intelligent sports sensors and have been widely applied in routine exercise, sports health and rehabilitation, and competitive sports. The H-TENG device, which consists of one or more types of polymers, incorporates hydrogel materials with various characteristics, including but not limited to conductivity, stability, and biocompatibility, as detailed in Section 3 of this review. These attributes confer a distinct advantage to H-TENGs when used as a human motion monitoring device, highlighting their significant potential for applications in intelligent sports. H-TENGs are capable of collecting diverse sports data, thereby facilitating a comprehensive understanding of human performance in athletic activities. Furthermore, motion data can be enhanced through machine learning techniques to improve human–computer interaction, enabling universal data collection and personalized feedback. The recent research on the performance of H-TENGs in the field of intelligent sports has been summarized in Table 3.

### 4.1. Energy Harvesting and Sensing from Daily Motion

Various modes of human movement can generate mechanical energy that can be harvested, including running, walking, joint flexion and extension, facial expression changes, and even arterial pulsations. This energy is sustainable and readily available, unaffected by time, location, or other constraints.

**Figure 4:**
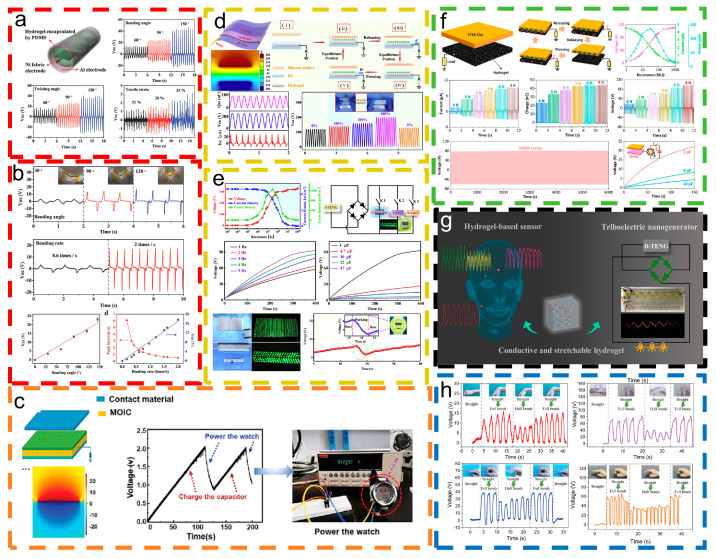
Application for daily motion energy harvesting and sensing. (**a**,**b**) The schematic structure of a tubular H-TENG and open-circuit voltage response for detecting joint motion versus bending angle. This content has been reproduced with permission from Ref. [48] (copyright 2016, John Wiley and Sons). (**c**) Charging of a commercial electronic watch using an MOIC-TENG. This content has been reproduced with permission from Ref. [85] (copyright 2023, John Wiley and Sons). (**d**,**e**) The S-TENG is capable of monitoring both minor and strenuous movements. It can power a portable LED and a portable electronic watch. This content has been reproduced with permission from Ref. [86] (copyright 2020, Elsevier). (**f**) P-TENGs act as efficient energy harvesters. This content has been reproduced with permission from Ref. [87] (copyright 2023, AlP Publishing). (**g**) Hydrogel-based sensors are utilized for monitoring human movement and harvesting mechanical energy through D-ENGs. This content has been reproduced with permission from Ref. [88] (copyright 2022, American Chemical Society). (**h**) Voltage signals from the MH-TENG detected at the wrist, elbow, and fingers during sustained flexion. This content has been reproduced with permission from Ref. [89] (copyright 2021, John Wiley and Sons).

To collect these sustainable energy sources generated by the human body and to achieve motion monitoring, Xu et al. [48] introduced a flexible, recyclable, and environmentally friendly hydrogel-based triboelectric nanogenerator (hydrogel TENG). Figure 4a,b demonstrate that the tubular hydrogel TENG effectively harvests mechanical energy from diverse human motions, enabling it to power 20 commercial white LEDs concurrently. Additionally, the system functions as a self-powered sensor for detecting human elbow joint movements. Long et al. [85] designed an organic gel ionic conductor (MOIC) prepared through a self-polymerization reaction. As shown in Figure 4c, the high-performance triboelectric nanogenerator (MOIC-TENG) constructed using MOIC can charge capacitors and power commercial electronic watches. Hydrogel-based devices function as highly stretchable triboelectric nanogenerators (STENGs), effectively differentiating between subtle and intense human movements while efficiently harvesting energy. As shown in Figure 4d,e, Sun et al. [86] prepared stretchable conductive hydrogels through a hybrid double-network method, capable of lighting LEDs and possessing sustainable energy. Figure 4f illustrates a hybrid conductive polyvinyl alcohol, cotton paper, graphene oxide, and MXene-based hydrogel (PCGM) designed by Zhang et al. [87]. PCGM-based strain sensors and triboelectric nanogenerators (P-TENGs) can be applied to human joints and facial skin for monitoring body posture and expressions while also functioning as effective energy harvesters.

Figure 4g presents a wearable strain sensor developed by Dong et al. [88] that can be used to monitor both subtle and vigorous human body movements. The hydrogel-based sensor functions as a deformable triboelectric nanogenerator (D-TENG) for mechanical energy harvesting. Luo et al. [89] presented an MXene/polyvinyl alcohol (PVA) hydrogel triboelectric nanogenerator (MH-TENG) for monitoring body movements. As shown in Figure 4h, the MH-TENG can stably and sensitively respond to continuous motion changes in the form of voltage signals. When the MH-TENG was attached to the finger of another subject, the same results were obtained.

In conclusion, the innovative design of H-TENGs enables efficient harvesting of mechanical energy generated during motion, thereby achieving self-powered functionality. Additionally, as high-performance sensors, they can accurately capture and record electrical signal data resulting from human movements. These data not only facilitate a comprehensive understanding of users’ physical conditions but also provide a robust foundation for the development of scientifically informed exercise regimens.

### 4.2. Human Sports Health and Rehabilitation

In addition to its capabilities in energy harvesting from routine human activities and data acquisition, the H-TENG significantly advances the domain of sports healthcare and rehabilitation. When compared to conventional devices, H-TENGs offer numerous advantages, including reduced cost, compact size, enhanced comfort, and innovative power supply methods. Furthermore, its integration with other electronic devices for assisted therapy presents novel opportunities for improving sports healthcare and rehabilitation outcomes.

**Figure 5:**
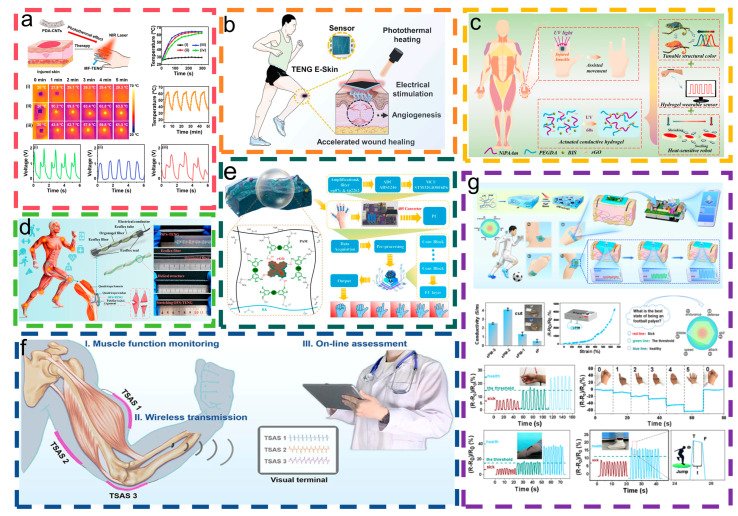
Human sports health and rehabilitation. (**a**) MF-TENG photothermal treatment simulation charts for finger flexion detection in healthy and injured subjects before and after photothermal treatment. This content has been reproduced with permission from Ref. [51] (copyright 2021, American Chemical Society). (**b**) The TENG E-skin patch: NIR photothermal heating, electrical stimulation, sensing, and wound healing promotion. This content has been reproduced with permission from Ref. [53] (copyright 2022, Elsevier). (**c**) Stretchable conductive composite hydrogel solutions for joint rehabilitation [90]. (**d**) Applications and architecture of OFS-TENG. This content has been reproduced with permission from Ref. [71] (copyright 2021, American Chemical Society). (**e**) An online rehabilitation training system based on gesture recognition. This content has been reproduced with permission from Ref. [91] (copyright 2023, American Chemical Society). (**f**) TSAS can obtain functional signals and analyze the biceps and triceps muscles. This content has been reproduced with permission from Ref. [52] (copyright 2021, American Chemical Society). (**g**) Schematic diagram of wireless sensors designed to monitor a soccer player’s health status. This content has been reproduced with permission from Ref. [88] (copyright 2023, Elsevier).

Targeting joint site recovery and wound healing promotion and addressing the problem of triboelectric nanogenerators affecting work time due to wear and unpredictable damages, Yang et al. [51] developed a single-electrode multifunctional triboelectric nanogenerator (MF-TENG) that possesses rapid self-healing abilities, good health monitoring capabilities, and strong photothermal performance. Figure 5a demonstrates that the MF-TENG facilitates photothermal therapy when exposed to near-infrared laser irradiation. Elevated temperatures enhance microcirculation and alleviate pain in injured areas, aiding the recovery of joint mobility. Du et al. [53] introduced an E-skin patch utilizing a single-electrode TENG that combines electrical stimulation and photothermal heating to detect motion and enhance wound healing. Figure 5b demonstrates that integrating photothermal heating with real-time electrical stimulation in the E-skin patch significantly enhances angiogenesis, collagen deposition, and re-epithelialization, thus expediting tissue regeneration and wound healing. Shao et al. [90] proposed a new type of conductive structural colored composite hydrogel, designed for robotic joint rehabilitation skin. As shown in Figure 5c, this wearable hydrogel film contains conductive reduced graphene oxide, capable of accurately sensing and monitoring human motion information. This stretchable conductive composite hydrogel solution provides an innovative solution for joint rehabilitation.

Implantable sensors that facilitate real-time health monitoring and assist with training are essential for addressing muscle and ligament injuries resulting from sports activities or diseases. As shown in Figure 5d, Sheng et al. [71] manufactured a self-powered, suturable, implantable ligament strain sensor by using an organic gel/silicon fiber helical triboelectric nanogenerator (OFS-TENG), which has high stability and ultra-stretchability. This design successfully realizes the real-time monitoring of human ligaments and muscles, providing a solution for the rapid diagnosis of human injuries.

Gesture recognition systems are a microcosm of modern intelligent methods for rehabilitation training. Li et al. [91] proposed an innovative gesture recognition system. Figure 5e illustrates the integration of hydrogel strain sensors and machine learning to enhance finger rehabilitation training. By leveraging machine learning techniques, the prospects for patient recovery have been enhanced, and the healthcare applications of the gesture recognition system have been strengthened. Evaluating muscle function is crucial for determining health status, assessing motor abilities, and guiding rehabilitation efforts. Wang et al. [52], building upon this foundation, synthesized an ionic hydrogel with a large strain range and rapid self-healing capabilities. Based on the coupling principle of contact electrification and electrostatic induction, they introduced a stretchable, self-healing, skin-attachable active sensor (TSAS). As shown in Figure 5f, the TSAS can simultaneously obtain functional signals from the biceps and triceps. The signals can also be wirelessly transmitted to a terminal for analysis. The TSAS features high sensitivity, reliability, ease of use, and low cost, and it is expected to become a new method for real-time assessment of muscle function and rehabilitation training in the next generation.

In intense competitions, the risk of injury for athletes is higher. Minimally invasive surgery, with its small incisions and quick recovery, remains the preferred choice for athletes. Knowing in advance whether an athlete is recovering within the expected time frame is beneficial for helping them return to the field and regain their performance as soon as possible. Sun et al. [92] created a medical hydrogel designed for both minimally invasive procedures and wearable diagnostics. As shown in Figure 5g, the hydrogel has excellent sensing capabilities and is capable of truly recording athletic motion characteristics in real time. It can wirelessly transmit information to the terminal via Bluetooth, enabling precise assessments of health recovery. This study pioneers the integration of hydrogels with wearable devices in minimally invasive treatments, paving the way for enhanced treatment efficiency and precise rehabilitation monitoring.

Inappropriate exercise techniques and uncertain exercise environments are significant contributors to sports injuries. Athletes who engage in high-intensity training or competitions without adequate technical guidance, or who train in unsuitable environments, are at an increased risk of injury. The H-TENG, an innovative self-powered sensor, has demonstrated considerable potential in this context. It not only collects real-time sports data but also analyzes these data to predict potential injury risks. By monitoring athletes’ physiological parameters and movement patterns, H-TENGs can detect abnormal exercise behaviors, prompting athletes to modify their exercise methods or take necessary rest. Additionally, the specialized design of H-TENGs enables them to address known sports injuries, facilitating targeted healthcare and rehabilitation treatments.

### 4.3. Intelligent Monitoring of Sports

With the development of the intelligent sports field, incorporating advanced sports data into intelligent devices for professional training teams has become an irreversible trend. Tracking athletes’ data in real time during training and competitions can optimize training programs and enhance competitive performance, providing solid data support for scientific training. H-TENGs have introduced a new path for personalized customization of intelligent sports equipment. Based on the specific needs of different competitive sports, excellent performance-based hydrogel sensors can be flexibly designed to provide auxiliary support for athletes in various competitive scenarios, promoting the advancement and integration of sports technology.

#### 4.3.1. Intelligent Monitoring of Summer Sports with Specific Equipment Requirements

**Figure 6:**
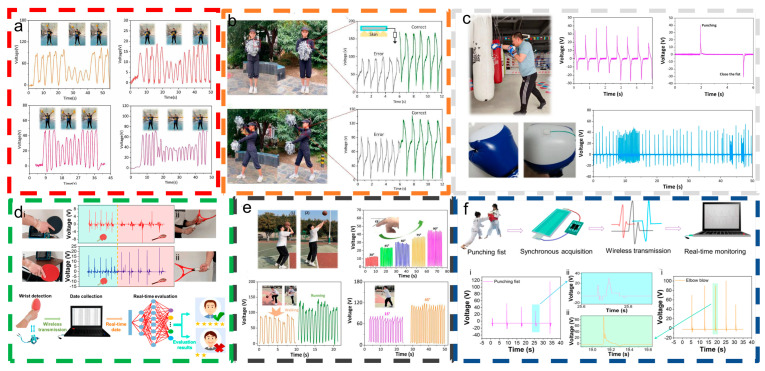
Tool-dependent intelligent monitoring applications for summer sports. (**a**) LP-TENG output voltage signals during various cheerleading maneuvers to monitor body movement [93]. (**b**) Correct and incorrect cheerleading poses and corresponding CF-TENG output voltage signals [94]. (**c**) H-TENGs mounted on boxing gloves for boxing exercises, the relationship between Isc and Voc under different forces, and the monitoring information obtained from continuous strikes [95]. (**d**) Implementation of the FL-TENG wireless system for optimizing attacking techniques in table tennis (i) and tennis (ii) [96]. (**e**) The electrical output of PPL-TENG at different draw amplitudes during the shooting and dribbling of basketball players. This content has been reproduced with permission from Ref. [54] (copyright 2023, AlP Publishing). (**f**) FL-TENG taekwondo match monitoring foul action and surveillance system [97].

Sports such as cheerleading, basketball, and table tennis require participants to hold equipment such as balls and rackets to complete their movements. Considering monitoring these specific pieces of equipment is worthwhile. To tackle the challenge of data monitoring during stretching exercises, Wang et al. [93] reported a LiCl/PVA hydrogel triboelectric nanogenerator (LP-TENG) characterized by high electrical output and simple fabrication. In Figure 6b, the LP-TENG is installed on human fingers, elbows, and shoulders to detect their movements and demonstrate output voltage signals under different conditions. In a related study, Gai et al. [94] employed a dual-network polymer ionic conductor, specifically a carboxymethyl chitosan (CMCh)–Fe hydrogel triboelectric nanogenerator (CF-TENG). The CF-TENG device functions as a self-powered smart sensor for monitoring arm-extension movements when configured as an exercise belt. As shown in Figure 6a, the conductive hydrogel-based CF-TENG self-powered elastic training belt sensor is used to monitor cheerleading practice movements. As such, CF-TENGs and LP-TENGs have been shown to have potential applications in future sports monitoring.

Boxing has emerged as one of the most popular sports globally, thanks to its diverse positive effects and extensive cultural influence. Consequently, the development of monitoring devices, particularly wearable sports monitoring technologies, has become increasingly important. Yang et al. [95] introduced a self-healing hydrogel triboelectric nanogenerator (H-TENG) designed for biomechanical energy harvesting and boxing training monitoring. Figure 6c demonstrates that the H-TENG functions independently as a force-detection sensor, exhibiting voltage variations in response to different pressures. It can also be installed on boxing gloves to monitor boxing movement information, realizing a self-powered sports sensor.

While people pursue a healthy and active lifestyle, injuries are inevitable. To prevent joint injuries caused by incorrect movements, Mao et al. [96] developed a wireless intelligent motion-correction system using an FL-TENG with hydrogel electrodes to improve durability and stability. As illustrated in Figure 6d, the system records voltage signals generated by both standard and incorrect table tennis and tennis ball swinging techniques. This wireless intelligent error-correction system integrates triboelectric nanogenerator technology with a wireless intelligent host for signal processing and visualization. It aims to detect and correct improper actions during the early stages of motor skill acquisition and refine technical actions during the broader phase of motor skill development, thereby preventing sports injuries caused by incorrect technique.

The advancement or enhancement of basketball technology necessitates effective monitoring of basketball movements. Deng et al. [54] developed a PVA/PTT/LiCl hydrogel triboelectric nanogenerator (PPL-TENG) for self-powered basketball movement monitoring. Figure 6e demonstrates that PPL-TENGs, following experimental testing, can identify human joint and gait characteristics in basketball movements, providing auxiliary support in the sport.

Athletes and coaches frequently express concerns regarding the fairness and impartiality of competition referees. To address this problem, Sun et al. [97] introduced a lightweight and flexible triboelectric nanogenerator (FL-TENG) utilizing hydrogel electrodes. Figure 6f illustrates the use of the FL-TENG as a self-powered sensor on taekwondo protective gear, facilitating athlete performance monitoring and promoting fair competition. Additionally, the FL-TENG can wirelessly power miniature devices to transmit motion data in real time during competitions.

In the aforementioned studies, alongside the monitoring of athletes, enhancements have enabled the monitoring of the equipment they utilize. This advancement facilitates the acquisition of essential data throughout the athletic process. Despite the relative scarcity of such research, it offers novel perspectives and potential applications for the advancement of future sports monitoring technologies.

#### 4.3.2. Intelligent Monitoring of Summer Sports Without Specific Equipment Requirements

Sports that do not necessitate specific equipment pertain to activities where participants have the flexibility in attire, such as running, diving, and computer gaming. This section focuses on the pertinent research related to these activities and offers considerations for broader applications.

**Figure 7:**
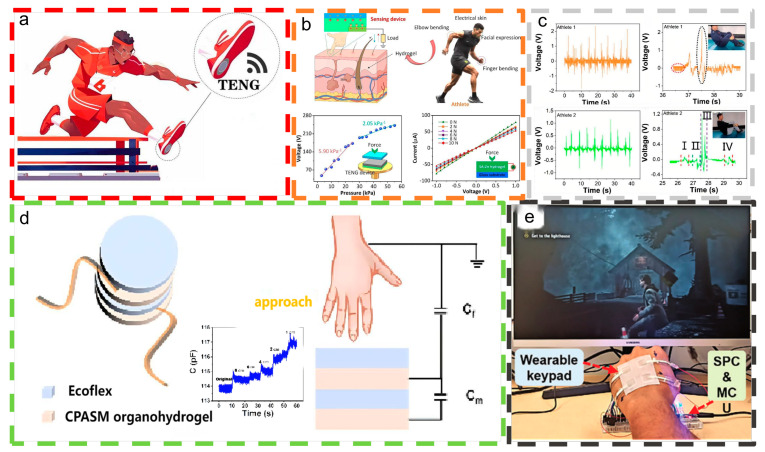
Intelligent monitoring of summer sports without specific equipment needs. (**a**) Utilization of a DES-TENG for monitoring stride frequency in hurdle races. This content has been reproduced with permission from Ref. [55] (copyright 2024, Elsevier). (**b**) F-Zn hydrogel-based strain sensors and triboelectric nanogenerators for monitoring running training. This content has been reproduced with permission from Ref. [98] (copyright 2022, Elsevier). (**c**) Flexible nanogenerators for monitoring 301 C technology in diving [99]. (**d**) A schematic diagram illustrating a proximity sensor and the variation in capacitance values of a sensing hand at varying distances. This content has been reproduced with permission from Ref. [100] (copyright 2023, Elsevier). (**e**) A self-powered wearable keyboard developed as a human-machine interface (HMI) for controlling the computer game ‘Alan Wake’. This content has been reproduced with permission from Ref. [101] (copyright 2023, John Wiley and Sons).

To satisfy the growing health needs by improving the accuracy and practicality of daily motion monitoring, Zhu et al. used conductive hydrogels and TENGs [55] to create a self-powered motion sensor with high precision, high stability, and high output power. Figure 7a illustrates the application of the DES-TENG in track and field events, like triple jump and hurdles, which necessitate high-voltage sensing monitoring. The research used schematic drawings instead of actual application scene images and did not provide actual application videos. Therefore, the practical application of these sensors in track and field sports scenarios is a new direction for research. Yang et al. [98] developed a flexible amphiphilic ion-conducting hydrogel (F-Zn) for use in strain sensors and friction nanogenerators (F-TENGs). As depicted in Figure 7b, this F-Zn hydrogel-based strain sensor acts like electronic skin, effectively tracking various running postures. By integrating two sensors with distinct functionalities, the system can monitor running movements, laying the groundwork for the development of a multifunctional hydrogel sensor for runners.

While people pursue healthy sports activities, they should also be mindful of sports injuries caused by incorrect movements. To address the aforementioned problem, Zhu et al. [99] developed a flexible, stretchable, self-healing composite nanogenerator for monitoring human motion. Figure 7c illustrates its application in monitoring multidimensional human body movements, including bending, twisting, and rotating, similar to the land training for the 301C diving technique.

The study of noncontact capacitive sensors is a very interesting direction for research. Zhou et al. [100] successfully prepared a stretchable, self-adhesive, freeze-resistant, and moisturizing ionic conductive organohydrogel. Figure 7d demonstrates the organohydrogel’s superior energy-harvesting performance when integrated with an Ecoflex elastomer in a TENG assembly. It ensures stable performance in proximity sensing. This device should be further researched to provide good assistance in sports scenarios involving the visually impaired, such as designing special paths to allow visually impaired people to engage in running exercises.

In order to achieve control of computer games through a wearable interface system, Rahman et al., in their study [101], incorporated ZIF-8 nanoparticles into a LiCl-based hydrogel to create highly stretchable and durable electrodes for wearable TENGs. Figure 7e illustrates the development of a wearable keyboard designed for controlling computer games. The application of wearable TENGs in e-sports is another emerging research direction.

Based on the aforementioned studies, it is clear that sports not requiring specialized equipment are distinguished by the lack of necessity for special gear and the ease of participation, thereby enhancing accessibility for diverse populations. In such sports, the H-TENG is employed to monitor movement accuracy and to detect proximity or contact. Future research should concentrate on the practical applications of the H-TENG in real-world contexts, encompassing more detailed use cases, long-term monitoring outcomes, and its adaptability across various demographic groups.

#### 4.3.3. Intelligent Monitoring of Winter Sports

Winter sports encompass a range of activities performed in icy and snowy environments, including disciplines such as skiing, ice skating, curling, and ice hockey. The monitoring of these sports necessitates operation within low-temperature settings, requiring participants to utilize cold-resistant equipment. This presents distinct challenges to the monitoring process, including issues related to low temperatures and harsh environmental conditions.

**Figure 8:**
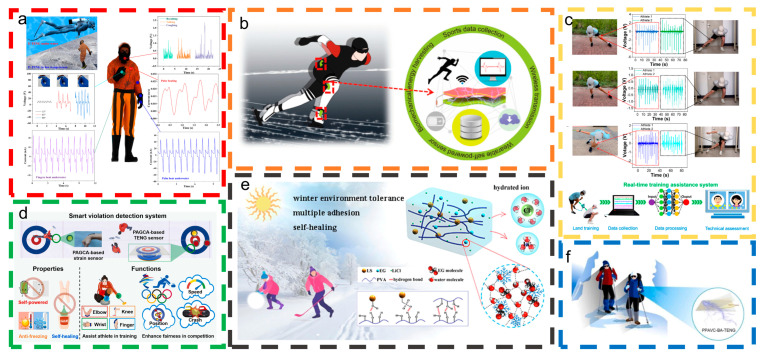
Intelligent monitoring applications for ice and snow sports. (**a**) E-TENG-based self-powered electronic devices function effectively in cold and underwater environments. This content has been reproduced with permission from Ref. [102] (copyright 2024, Springer Nature). (**b**) Application scenarios of MS-TENGs [103]. (**c**) Utilization of SD-TENGs in land training for speed skating [104]. (**d**) Schematic diagram of the violation-detection system for curling events and its performance and functionality. This content has been reproduced with permission from Ref. [56] (copyright 2024, Elsevier). (**e**) A self-repairing hydrogel with adhesive properties as a winter sports sensor prepared by the one-pot method in this study. This content has been reproduced with permission from Ref. [105] (copyright 2023, Elsevier). (**f**) The PPAVC-BA-TENG exhibits low-temperature electrical output performance. This content has been reproduced with permission from Ref. [106] (copyright 2024, Elsevier).

Intelligent monitoring of winter sports faces unique challenges, especially the stability of H-TENG devices in low-temperature environments. Wu et al. [102] proposed a solution to this problem by introducing an antifreezing eutectic gel that maintains the stability and conductivity of the TENG under harsh environmental conditions. As shown in Figure 8a, the E-TENG sensor can identify walking patterns, throat vibrations related to speech, resting pulse rates, and the movement of small or large joints in the body in cold and underwater environments. This device has the capability to sense human signals under harsh environmental conditions (such as low temperatures, underwater, and even in the air) and it can be further researched for safety assurance and motion monitoring in extreme sports such as ski mountaineering, wingsuit flying, and surfing.

Effectively evaluating speed skaters’ technical movements is crucial for improving athletes’ performance. Lu et al. [103] prepared a self-powered, portable microstructured triboelectric nanogenerator (MS-TENG). Figure 8b demonstrates the application of the MS-TENG in monitoring sports training and conducting big data analysis for speed skating and other athletic activities. In addition, Lu et al. [104] conducted a study to observe alterations in skaters’ joints and joint chains during land training, preparing a stable and durable triboelectric nanogenerator (SD-TENG), which has good mechanical properties and triboelectric performance. Figure 8c illustrates the voltage output and action completions during speed skaters’ land training, including knee joint flexion and ankle joint angle variations, alongside the operation mode of the SD-TENG-based wireless intelligent land auxiliary training system.

In order to make quick and fair judgments in the statistics and analysis of sports competitions, Tian et al. [56] developed a PACCA-based self-powered, self-healing smart violation-detection system for curling sports events. Figure 8d shows multiple TENG sensors in the system that are assembled to determine rule violations, detect the speed and exact location of the curling stone, and distinguish collisions during curling matches.

To enable sensing in challenging outdoor conditions in winter, Yang et al. [105] prepared an adhesive self-healing hydrogel for winter sports sensors. Figure 8e illustrates the synthesis of a multifunctional hydrogel using a one-pot method. LiCl was used to entangle PVA chains, forming the hydrogel skeleton, while the addition of LS and EG imparted the material with enhanced conductivity, water absorption, and self-healing capabilities, and resistance to UV radiation, freezing, and drying. This hydrogel device is fully capable of completing human motion monitoring in snowy environments and can provide functions such as motion detection, human safety positioning, and information transmission for winter sports like alpine skiing.

Currently, hydrogel-based flexible strain sensors and portable TENGs often face challenges such as limited stretchability and flexibility, inadequate mechanical performance, poor tolerance to low temperatures, and insufficient power output. Lei et al. [106] developed a single-electrode TENG using PPAVC-BA hydrogel as the electrode, which demonstrated good energy collection capability. Figure 8f illustrates that it is capable of maintaining good electrical output performance in both low (−50 °C)- and high (80 °C)-temperature environments. The PPAVC-BA-TENG shows significant promise for use in motion monitoring within ice and snow sports.

The current body of literature on the application of H-TENGs for detection purposes in summer and winter sports remains limited. Existing research predominantly concentrates on the use of H-TENGs for collecting joint movement data from athletes and acquiring data through attachment to sports equipment. This data collection facilitates the development of intelligent sports systems that offer users precise action assessments and personalized training programs, thereby enhancing the efficiency of sports monitoring. The swift advancements in intelligent sports monitoring technology provide sophisticated training support and safety assurances for athletes. In both summer and winter sports, the ongoing improvements in sensor design and material performance contribute to the increased accuracy and practicality of sports monitoring. These advancements not only satisfy the growing demand for scientifically informed and health-oriented sports practices but also introduce innovative concepts and opportunities for the future evolution of sports technology. As these technologies become more widespread, they are anticipated to be applied across a broader spectrum of sports disciplines, thereby fostering the continuous progression of sports science and intelligent monitoring.

### 4.4. Human–Machine Interfaces in Intelligent Sports

The human–machine interface (HMI) encompasses the various modalities through which humans engage with machines or systems, serving as an integral component of contemporary technological frameworks. With the ongoing progression of technological advancements, the design and material selection of HMIs are undergoing significant evolution to address the escalating complexity of user requirements and application contexts. Hydrogel materials, characterized by their flexibility, conductivity, and biocompatibility, are particularly well suited for the development of HMIs, thereby driving innovation in the realm of intelligent sports equipment and systems.

**Figure 9:**
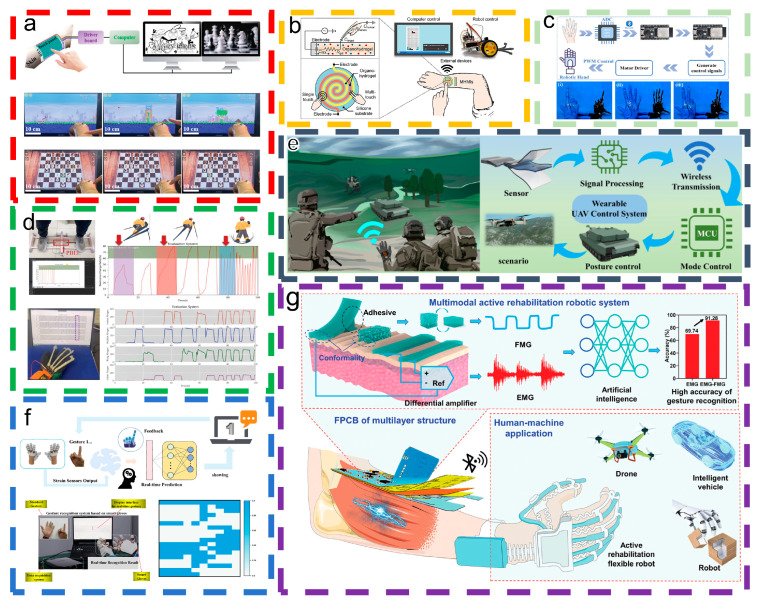
Uses of H-TENGs at intelligent motion human–machine interfaces. (**a**) Epidermal touch screen-operated computer game. This content has been reproduced with permission from Ref. [57] (copyright 2022, John Wiley and Sons). (**b**) Schematic of a multifunctional HMI based on an antidrying organic hydrogel. This content has been reproduced with permission from Ref. [58] (copyright 2022, Elsevier). (**c**) Flowchart of the operation of a glove-based HMI system, (i) 2-finger gesture, (ii) Palm open, (iii) 3-finger gesture. This content has been reproduced with permission from Ref. [107] (copyright 2024, Elsevier). (**d**) Simulation of skiing training assessment and finger movement training and assessment. This content has been reproduced with permission from Ref. [59] (copyright 2024, John Wiley and Sons). (**e**) Schematic of a control system based on the GE-TENG integrated tank model. This content has been reproduced with permission from Ref. [108] (copyright 2024, Elsevier). (**f**) Schematic illustration of the control system employed for real-time gesture recognition and feedback, featuring smart gloves, neural network algorithms, and accompanying physical diagrams [109]. (**g**) Workflow for active rehabilitation or other HMI application systems. This content has been reproduced with permission from Ref. [110] (copyright 2023, John Wiley and Sons).

Aiming to solve the problem of traditional touch panels’ rigid and fragile electrodes, Guo et al. [57] presented a TENG-based multifunctional capacitive touch screen that serves as a human–machine interaction interface, maintaining functionality in high-stretch and sub-zero conditions. As shown in Figure 9a, the touch panel can operate the computer games “Angry Birds” and “Chess” through finger touch. Wu et al. [58], addressing the limitation of flexible touch panels being restricted to single-touch operations, introduced a robust, wearable multifunctional HMI (MHMI) utilizing a transparent, ionic conductive, and resilient organic hydrogel, capable of precise and sensitive detection of single or multiple touches. Figure 9b depicts the sensing principle and circuit diagram of the organic hydrogel flexible ionic conductor. Multitouch recognition enhances MHMIs by enabling seamless device switching and multifunction selection, thereby improving operability.

**Table 3:** Performances of H-TENGs in intelligent sports.

Components of Polymer Networks	Conductive Materials	Cycles	Skin Contact	Short-Circuit Current	Open-Circuit Voltage	Application	Ref.
PVA/sodium borate	PDA-CNTs	1000	√	7.89 μA	38.57 V	Photothermal treatment	[51]
PVA	LiCl	10^5^	√	1.42 μA	78.4 V	Analyze muscle signals	[52]
PPy/F127	PPy	45,000	√	-	75 V	E-skin	[53]
P(AM-MA)-PEI	MA	4000	√	2.8 μA	225 V	Hurdle races	[55]
MDHS/HEMA/AA	LiCl	15,000	√	5.1 μA	123 V	Touch screen	[57]
Organogel/silicone	LiTFSI	30,000	Implantable	-	2 V	Real-time monitoring	[71]
PAM	NaCl	1800	√	7 μA	160 V	Charging	[85]
PAM/gelatin	PEDOT:PSS	1200	√	26.9 μA	383.8 V	Charging/monitoring	[86]
PVA/CP	GO and MXene	60,000	√	10.36 μA	229.85 V	Energy harvesting	[87]
PAAM/PAA	PEDOT:PSS/GR	1000	√	0.8 μA	141 V	Energy harvesting/monitoring	[88]
PVA	MXene	-	√	270 nA	230 V	Joint motion detection	[89]
CMCh-Fe	FeCl_3_	15,000	√	-	-	Cheerleading	[94]
LPG-KCL	KCl	15,000	-	0.72 μA	90 V	Boxing	[95]
PAAM	LiCl	2800	√	-	514 V	Taekwondo	[97]
SA/AM/AA	F-Zn	30,000	√	9.16 μA	245.54 V	Running monitoring	[98]
CPASM	LiCl	-	NC	-	102 V	Proximity sensor	[100]
PAAm-co-HEA	ZIF-8/LiCl	50,000	√	-	238 V	Wearable keyboard	[101]
SL/AA/APS	FeCl_3_	10,000	√	1.54 μA	776 V	Cold/underwater	[102]
GNOH	NaCl	-	√	3.8 μA	142 V	Integrated tank model	[108]

Fu et al. [107] aimed to stabilize HMI applications in low-temperature settings by developing a dual-network hydrogel composed of agar and PAAm, doped with antifreeze proteins and sodium chloride, distinguished by its excellent antifreeze properties. This hydrogel is ideal for daily use and can be integrated into glove-based HMI systems for smart monitoring. Figure 9c illustrates that the Na-Agar/PAAm-AFP hydrogel effectively operates as a sensor in HMI systems, enabling accurate finger movement tracking and enhancing intelligent human–robot interactions. To address the challenge of hydrogels losing moisture and freezing in cold conditions, Liu et al. [59] introduced the ionic co-hybrid hydrogel PBLL, which exhibits a humidity-adaptive function. Figure 9d illustrates how integrating PBLL hydrogel with sports training monitoring creates a real-time human–computer interaction system for evaluating sports training and rehabilitation.

In the study of hydrogel sensors for electrical and mechanical properties, Xie et al. [108] developed a transparent and highly conductive gelatin–NaCl organic hydrogel (GNOH) with long-term stability. A stretchable and durable gelatin Ecoflex triboelectric nanogenerator (GE-TENG) was developed by integrating an organic hydrogel with the Ecoflex elastomer. Figure 8e illustrates a self-powered smart sensing system for human–machine interaction using GE-TENGs (GSIS). This wearable intelligent system combines signal acquisition, processing, and output to control tank models and robotic arms.

With the aim of enriching the life experience of hearing-impaired people and reducing the cost of their communication with the world, Lu et al. proposed a low-cost, high-efficiency gesture language recognition and feedback system based on a 3D-printed glove, an SF-hydrogel strain sensor array, a data acquisition card, a signal processing module, and a gesture recognition interface [109]. Figure 9f illustrates the gesture recognition and feedback system, comprising a strain sensor glove for capturing finger movements, a printed circuit board for signal preprocessing, an NI data acquisition card linked to a PC for data collection, and deep learning-based analysis system for signal recognition. The real-time demonstration interface employs a neural network algorithm to identify the final gesture and present feedback signals.

By integrating advanced sensing technology, optimized algorithms, and ergonomically designed materials, Wang et al. [110] developed a versatile multimodal human–machine interface featuring a hydrogel-based sensor and a custom flexible printed circuit board, focusing on enhancing sensing technology, optimizing algorithms, and employing ergonomically designed materials. Figure 9g illustrates a schematic diagram of a wearable HMI enabled by hydrogel-based electromyography (EMG) and pressure sensors, along with the corresponding artificial intelligence (AI)-assisted smart active rehabilitation robot system. The hydrogel’s composition and structural design enable precise and stable EMG and FMG signal collection, aiding AI-assisted decoding.

Due to their inherent flexibility, electrical conductivity, and biocompatibility, hydrogels are well suited for the development of next-generation human–machine interfaces (HMIs). The hydrogel-based triboelectric nanogenerator (H-TENG) not only facilitates the monitoring of human motion but also addresses the challenge of maintaining a continuous power supply. Furthermore, it is capable of creating touch-sensitive interfaces that produce varied tactile feedback in response to finger swipes, presses, or gestures. This technology is expected to play a pivotal role in numerous applications, particularly in the realm of smart motion, which necessitates high sensitivity and adaptability.

## 5. Existing and Future Challenges

### 5.1. Stability and Durability of Hydrogel Materials

In high-intensity sports scenarios, H-TENGs need to withstand frequent stretching, bending, and friction operations. Mechanical fatigue, fracture, or surface damage may occur, leading to a gradual decline in sensor performance; these are key factors limiting their application. The main component of ordinary hydrogel materials is the polymer network with high water content, which can easily lose water or freeze under extreme conditions (such as high temperatures, low temperatures, or dry or high-humidity conditions), resulting in a significant decline in mechanical and electrical properties. Although special materials can be integrated into hydrogel materials during their preparation to enhance performance in extreme environments, previous studies have not reported hydrogel materials that are acceptable for use in all extreme environments.

### 5.2. Bottleneck in Energy Output Performance of H-TENGs

As is well known, TENGs generate electrical signals by collecting the mechanical energy generated by the human body and achieve human motion monitoring through analysis of the electrical signals. H-TENGs also have a place in human motion monitoring, but their output performance is usually lower than that of traditional solid material TENGs. The soft surface of the hydrogel may lead to an insufficient friction contact area, which limits the efficiency of charge generation. Due to the low frictional charge density and the limited surface charge transfer efficiency of hydrogels, it is difficult to meet the demand of intelligent sports equipment for high power output. In intelligent sports applications, H-TENGs need to balance flexibility, transparency, biocompatibility, and high output performance. However, there are often contradictions between these characteristics.

### 5.3. Challenges in Preparation Process and Large-Scale Application of H-TENGs

Intelligent sports devices typically require designs that are lightweight, compact, and highly integrated. However, the flexibility and stretchability of H-TENGs may be limited during the integration process, and technological breakthroughs are still needed to achieve device miniaturization and efficient integration without affecting performance. The preparation of H-TENGs typically involves multistep chemical synthesis and fine processing. These processes are complex and costly, which is not conducive to large-scale production. In addition, the sensitivity to environmental conditions such as temperature and humidity during the preparation process also increases the difficulty of the process. In batch production, the mechanical and electrical properties of hydrogels may fluctuate significantly due to small changes in preparation conditions, which highlights the need for more stringent requirements in practical applications. Ensuring the performance consistency of hydrogel materials and the repeatability of devices still needs further research.

### 5.4. Biocompatibility and Safety Issues of H-TENGs

With the popularization of intelligent sports equipment, the issue of waste disposal is increasingly receiving attention. The nondegradability of traditional hydrogel materials may cause environmental pollution, so the development of degradable H-TENG materials to reduce environmental impact is a matter of concern. Although hydrogels have good biocompatibility, they may cause allergy, irritation, or microbial infection when sensors are worn for a long time or are in contact with skin.

## 6. Conclusions and Perspectives

This paper reviews the advancements in H-TENG research within intelligent sports. It introduces the working principles and modes of H-TENGs. Then, it presents the characteristics of H-TENGs, including self-adhesion, stretchability, antibacterial properties, temperature resistance, and self-healing capabilities. Additionally, it examines H-TENG sensor applications in intelligent sports, focusing on energy harvesting and sensing, sports healthcare and rehabilitation, smart sports monitoring, and intelligent human–machine interfaces.

**Figure 10:**
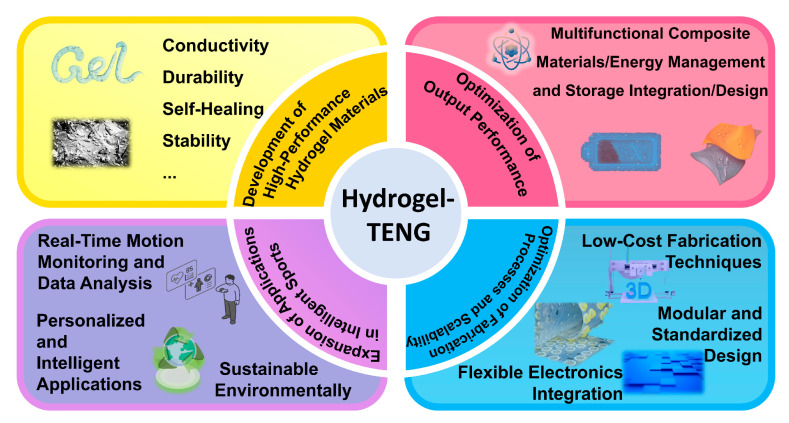
Perspectives.

H-TENGs have achieved notable advancements in intelligent sports. The high flexibility, stretchability, and excellent shape adaptability demonstrated by hydrogels undoubtedly provide H-TENGs with unique advantages for their application in this field, allowing them to better adapt to the diverse needs in intelligent sports. Although H-TENGs have shown remarkable effectiveness in the field of smart sports, it is still necessary to maintain the high level of attention given to the following four directions in order to explore their broader application prospects as shown in Figure 10: stability and durability of hydrogel materials, bottlenecks in output performance, challenges in fabrication and scalability, and biocompatibility and safety concerns.

### 6.1. Development of High-Performance Hydrogel Materials

Enhanced Environmental Adaptability: Developing hydrogel with antifreezing and antidrying properties, as well as high-humidity stability, is crucial. Additionally, dynamic network structures based on supramolecular chemistry can significantly enhance environmental adaptability.

Self-Healing and Durability Enhancement: Introducing dynamic chemical bonds (e.g., hydrogen bonds, ionic bonds, or dynamic covalent bonds) or nano-reinforcements (e.g., graphene or nanocellulose) can enable self-healing capabilities and enhance the mechanical properties of the hydrogel.

Efficient Ionic Conduction Mechanisms: Optimizing ionic conduction mechanisms in the hydrogel, such as designing high-ion-concentration solutions or incorporating ionic liquids, can improve charge transfer efficiency. Additionally, the low volatility and high conductivity of ionic liquids can significantly enhance the long-term stability of hydrogels.

### 6.2. Optimization of Output Performance

Interfacial Engineering Design: Surface modification or nanostructure design (e.g., micro/nano-patterning) can improve the charge transfer efficiency between the hydrogel and other materials.

Multifunctional Composite Materials: Developing composite materials that combine high flexibility, transparency, and output performance is essential. For instance, integrating hydrogels with conductive polymers (e.g., PEDOT:PSS), carbon nanomaterials (e.g., graphene or carbon nanotubes), or metallic nanoparticles can significantly enhance their conductivity and mechanical properties.

Energy Management and Storage Integration: Combining H-TENGs with efficient energy storage devices (e.g., supercapacitors or microbatteries) can enable efficient storage and management of the generated energy.

### 6.3. Optimization of Fabrication Processes and Scalability

Low-Cost Fabrication Techniques: Developing simple, efficient, and cost-effective fabrication processes for H-TENGs is critical. For example, 3D printing [111,112] or molding techniques can enable rapid prototyping of complex structures. Additionally, green chemical synthesis methods can reduce the environmental impact of fabrication.

Modular and Standardized Design: Promoting the modular design of H-TENG devices can simplify integration processes and improve production efficiency. For instance, standardized modules can enable quick assembly and replacement of different functional components.

Flexible Electronics Integration: Exploring the integration of flexible electronics with H-TENGs, such as using flexible circuit boards or printed electronics, can achieve efficient integration and multifunctionality in intelligent sports devices.

### 6.4. Expansion of Applications in Intelligent Sports

Real-Time Motion Monitoring and Data Analysis: Integrating H-TENGs into wearable devices can enable real-time monitoring of physiological parameters (e.g., heart rate, gait, and muscle activity) and motion data. For example, wireless transmission technology can transmit collected data to smart terminals for analysis and feedback.

Self-powered Energy Conversion System: H-TENGs can harvest mechanical energy from human movement, thus realizing continuous energy conversion. For example, H-TENGs are attached to human joints through multipoint control or embedded in sports shoes and sportswear through intelligent equipment integration, allowing for effective conversion of mechanical energy in the process of exercise into electrical energy.

Personalized and Intelligent Applications: Combining H-TENGs with artificial intelligence and big data technologies can enable personalized training guidance and intelligent feedback systems. For example, analyzing motion data can provide users with customized training recommendations and health management solutions.

Green and Sustainable Development: Promoting the use of biodegradable H-TENGs in intelligent sports applications can reduce the environmental impact of electronic waste. For instance, developing hydrogels based on natural polymers (e.g., chitosan or gelatin) can provide environmentally friendly and sustainable solutions.

## Figures and Tables

**Table 1:** Summary of materials, methods, and usage types of common hydrogels [44].

Method	Materials	Usage Type
Template	PAAm	Strain sensor
	Alginate and PAAm	Strain sensor
	PEDOT:PSS and PVA	Strain sensor
	PVA and HEC	Temperature sensor
	Agarose/SMF/PCF	Humidity sensor
Wet spinning	Sodium polyacrylate and SWCNT	Strain sensor
	SA/PANI/rGO	Strain sensor
	Chitosan and polypyrrole	Temperature sensor
	H-PAN and SP	pH-sensitive sensor
	GO and alginate	Actuator
Electrospinning	KGM/KC/PANI	Strain sensor
	TPU/P(NIPAM-ABP)	Actuator
	PAA and PCL	Actuator
Microfluidic spinning	Alginate and glycerol	pH-sensitive sensor
Draw spinning	P(AAm-co-AA)/Fe(III)	Humidity sensor

**Table 2:** Performance comparison between H-TENG and other sensors.

Sensor Type	Self-Powered	Cost of Materials	Extendibility	Environmental Suitability	Low-Frequency Micromotion Response	Ref.
Optical	No	Expensive	No	Bad	Bad	[3]
Resistive	No	Expensive	No	Good	General	[4]
Ultrasound	No	Expensive	No	Bad	General	[5]
MEMS	No	Expensive	No	Good	General	[6]
Piezoelectric	Yes	Expensive	No	Good	Good	[45]
Thermoelectric	Yes	Expensive	No	Bad	Bad	[46]
Photoelectric	Yes	Expensive	No	Bad	Bad	[47]
Triboelectric	Yes	Inexpensive	No	Good	Good	[13]
H-TENG	Yes	Inexpensive	Yes	Good	Good	[44]

## Data Availability

No new data were created or analyzed in this study.

## References

[1] Kutcher J.S., McCrory P., Davis G., Ptito A., Meeuwisse W.H., Broglio S.P. (2013). What evidence exists for new strategies or technologies in the diagnosis of sports concussion and assessment of recovery?. Br. J. Sports Med..

[2] Ahmadi A., Mitchell E., Richter C., Destelle F., Gowing M., O’Connor N.E., Moran K. (2015). Toward Automatic Activity Classification and Movement Assessment During a Sports Training Session. IEEE Internet Things J..

[3] Leber A., Cholst B., Sandt J., Vogel N., Kolle M. (2019). Stretchable Thermoplastic Elastomer Optical Fibers for Sensing of Extreme Deformations. Adv. Funct. Mater..

[4] Wu L., Shi X.Y., Das P., Wu Z.S. (2023). Recent application progress and key challenges of biomass-derived carbons in resistive strain/pressure sensor. Sci. China Mater..

[5] Madore B., Preiswerk F., Bredfeldt J.S., Zong S.Y., Cheng C.C. (2021). Ultrasound-based sensors to monitor physiological motion. Med. Phys..

[6] Xu S., Rwei A.Y., Vwalika B., Chisembele M.P., Stringer J.S.A., Ginsburg A.S., Rogers J.A. (2021). Wireless skin sensors for physiological monitoring of infants in low-income and middle-income countries. Lancet Digit. Health.

[7] Fan F.-R., Tian Z.-Q., Wang Z.L. (2012). Flexible triboelectric generator!. Nano Energy.

[8] Wang Z.L., Chen J., Lin L. (2015). Progress in triboelectric nanogenerators as a new energy technology and self-powered sensors. Energy Environ. Sci..

[9] Parida K., Thangavel G., Cai G., Zhou X., Park S., Xiong J., Lee P.S. (2019). Extremely stretchable and self-healing conductor based on thermoplastic elastomer for all-three-dimensional printed triboelectric nanogenerator. Nat. Commun..

[10] Liu W., Wang Z., Wang G., Liu G., Chen J., Pu X., Xi Y., Wang X., Guo H., Hu C. (2019). Integrated charge excitation triboelectric nanogenerator. Nat. Commun..

[11] Xu W., Zheng H., Liu Y., Zhou X., Zhang C., Song Y., Deng X., Leung M., Yang Z., Xu R.X. (2020). A droplet-based electricity generator with high instantaneous power density. Nature.

[12] Luo J., Tang W., Fan F.R., Liu C., Pang Y., Cao G., Wang Z.L. (2016). Transparent and Flexible Self-Charging Power Film and Its Application in a Sliding Unlock System in Touchpad Technology. Acs Nano.

[13] Pang Y., Li J., Zhou T., Yang Z., Luo J., Zhang L., Dong G., Zhang C., Wang Z.L. (2017). Flexible transparent tribotronic transistor for active modulation of conventional electronics. Nano Energy.

[14] Yang H., Pang Y., Bu T., Liu W., Luo J., Jiang D., Zhang C., Wang Z.L. (2019). Triboelectric micromotors actuated by ultralow frequency mechanical stimuli. Nat. Commun..

[15] Choi J., Ghaffari R., Baker L.B., Rogers J.A. (2018). Skin-interfaced systems for sweat collection and analytics. Sci. Adv..

[16] Huang S., Liu Y., Zhao Y., Ren Z., Guo C.F. (2019). Flexible Electronics: Stretchable Electrodes and Their Future. Adv. Funct. Mater..

[17] He X., Xu T., Gu Z., Gao W., Xu L.-P., Pan T., Zhang X. (2019). Flexible and Superwettable Bands as a Platform toward Sweat Sampling and Sensing. Anal. Chem..

[18] He W., Wang C., Wang H., Jian M., Lu W., Liang X., Zhang X., Yang F., Zhang Y. (2019). Integrated textile sensor patch for real-time and multiplex sweat analysis. Sci. Adv..

[19] Yang Y., Song Y., Bo X., Min J., Pak O.S., Zhu L., Wang M., Tu J., Kogan A., Zhang H. (2020). A laser-engraved wearable sensor for sensitive detection of uric acid and tyrosine in sweat. Nat. Biotechnol..

[20] Gao W., Emaminejad S., Nyein H.Y.Y., Challa S., Chen K., Peck A., Fahad H.M., Ota H., Shiraki H., Kiriya D. (2016). Fully integrated wearable sensor arrays for multiplexed in situ perspiration analysis. Nature.

[21] Yang Y., Gao W. (2019). Wearable and flexible electronics for continuous molecular monitoring. Chem. Soc. Rev..

[22] Khan Y., Ostfeld A.E., Lochner C.M., Pierre A., Arias A.C. (2016). Monitoring of Vital Signs with Flexible and Wearable Medical Devices. Adv. Mater..

[23] Bao Z., Chen X. (2016). Flexible and Stretchable Devices. Adv. Mater..

[24] Wang Z., Guan X., Huang H., Wang H., Lin W., Peng Z. (2019). Full 3D Printing of Stretchable Piezoresistive Sensor with Hierarchical Porosity and Multimodulus Architecture. Adv. Funct. Mater..

[25] Xuan X., Yoon H.S., Park J.Y. (2018). A wearable electrochemical glucose sensor based on simple and low-cost fabrication supported micro-patterned reduced graphene oxide nanocomposite electrode on flexible substrate. Biosens. Bioelectron..

[26] Zhang B., Zhang L., Deng W., Jin L., Chun F., Pan H., Gu B., Zhang H., Lv Z., Yang W. (2017). Self-Powered Acceleration Sensor Based on Liquid Metal Triboelectric Nanogenerator for Vibration Monitoring. Acs Nano.

[27] Yoon J.H., Kim S.-M., Park H.J., Kim Y.K., Oh D.X., Cho H.-W., Lee K.G., Hwang S.Y., Park J., Choi B.G. (2020). Highly self-healable and flexible cable-type pH sensors for real-time monitoring of human fluids. Biosens. Bioelectron..

[28] Son D., Lee J., Qiao S., Ghaffari R., Kim J., Lee J.E., Song C., Kim S.J., Lee D.J., Jun S.W. (2014). Multifunctional wearable devices for diagnosis and therapy of movement disorders. Nat. Nanotechnol..

[29] Wang Z., Cong Y., Fu J. (2020). Stretchable and tough conductive hydrogels for flexible pressure and strain sensors. J. Mater. Chem. B.

[30] Seyedin S., Zhang P., Naebe M., Qin S., Chen J., Wang X., Razal J.M. (2019). Textile strain sensors: A review of the fabrication technologies, performance evaluation and applications. Mater. Horiz..

[31] Lee H.-R., Kim C.-C., Sun J.-Y. (2018). Stretchable Ionics—A Promising Candidate for Upcoming Wearable Devices. Adv. Mater..

[32] Yuk H., Lin S., Ma C., Takaffoli M., Fang N.X., Zhao X. (2017). Hydraulic hydrogel actuators and robots optically and sonically camouflaged in water. Nat. Commun..

[33] Lee J., Tan M.W.M., Parida K., Thangavel G., Park S.A., Park T., Lee P.S. (2020). Water-Processable, Stretchable, Self-Healable, Thermally Stable, and Transparent Ionic Conductors for Actuators and Sensors. Adv. Mater..

[34] Grigoryan B., Paulsen S.J., Corbett D.C., Sazer D.W., Fortin C.L., Zaita A.J., Greenfield P.T., Calafat N.J., Gounley J.P., Ta A.H. (2019). BIOMEDICINE Multivascular networks and functional intravascular topologies within biocompatible hydrogels. Science.

[35] Zhang Q., Liu X., Zhang J., Duan L., Gao G. (2021). A highly conductive hydrogel driven by phytic acid towards a wearable sensor with freezing and dehydration resistance. J. Mater. Chem. A.

[36] Lei Z., Wu P. (2021). Bioinspired Quasi-Solid Ionic Conductors: Materials, Processing, and Applications. Acc. Mater. Res..

[37] Yang C., Suo Z. (2018). Hydrogel ionotronics. Nat. Rev. Mater..

[38] An R., Zhang B., Han L., Wang X., Zhang Y., Shi L., Ran R. (2019). Strain-sensitivity conductive MWCNTs composite hydrogel for wearable device and near-infrared photosensor. J. Mater. Sci..

[39] Cai G., Wang J., Qian K., Chen J., Li S., Lee P.S. (2017). Extremely Stretchable Strain Sensors Based on Conductive Self-Healing Dynamic Cross-Links Hydrogels for Human-Motion Detection. Adv. Sci..

[40] Zhang Z., Wang L., Yu H., Zhang F., Tang L., Feng Y., Feng W. (2020). Highly Transparent, Self-Healable, and Adhesive Organogels for Bio-Inspired Intelligent Ionic Skins. ACS Appl. Mater. Interfaces.

[41] Ge G., Yuan W., Zhao W., Lu Y., Zhang Y., Wang W., Chen P., Huang W., Si W., Dong X. (2019). Highly stretchable and autonomously healable epidermal sensor based on multi-functional hydrogel frameworks. J. Mater. Chem. A.

[42] Xu J., Jing R., Ren X., Gao G. (2020). Fish-inspired anti-icing hydrogel sensors with low-temperature adhesion and toughness. J. Mater. Chem. A.

[43] Qin Z., Sun X., Yu Q., Zhang H., Wu X., Yao M., Liu W., Yao F., Li J. (2020). Carbon Nanotubes/Hydrophobically Associated Hydrogels as Ultrastretchable, Highly Sensitive, Stable Strain, and Pressure Sensors. ACS Appl. Mater. Interfaces.

[44] Du J.X., Ma Q., Wang B.H., Sun L.T., Liu L.M. (2023). Hydrogel fibers for wearable sensors and soft actuators. iScience.

[45] Juraij K., Shafeeq V.H., Chandran A.M., Vasudevan S., Mural P.K.S., Sujith A. (2023). Human body stimuli-responsive flexible polyurethane electrospun composite fibers-based piezoelectric nanogenerators. J. Mater. Sci..

[46] Dziadak B., Makowski L., Kucharek M., Jósko A. (2023). Energy Harvesting for Wearable Sensors and Body Area Network Nodes. Energies.

[47] Yan W., Ma C.B., Cai X.X., Sun Y.Y., Zhang G.L., Song W.X. (2023). Self-powered and wireless physiological monitoring system with integrated power supply and sensors. Nano Energy.

[48] Xu W., Huang L.-B., Wong M.-C., Chen L., Bai G., Hao J. (2017). Environmentally Friendly Hydrogel-Based Triboelectric Nanogenerators for Versatile Energy Harvesting and Self-Powered Sensors. Adv. Energy Mater..

[49] Guo H., Shi Y., Pan F., Zheng S., Chai X., Yang Y., Jiang H., Wang X., Li L., Xiu Z. (2023). Tough, stretchable dual-network liquid metal-based hydrogel toward high-performance intelligent on-off electromagnetic interference shielding, human motion detection and self-powered application. Nano Energy.

[50] Qin Y., Mo J., Liu Y., Zhang S., Wang J., Fu Q., Wang S., Nie S. (2022). Stretchable Triboelectric Self-Powered Sweat Sensor Fabricated from Self-Healing Nanocellulose Hydrogels. Adv. Funct. Mater..

[51] Yang D., Ni Y., Kong X., Li S., Chen X., Zhang L., Wang Z.L. (2021). Self-Healing and Elastic Triboelectric Nanogenerators for Muscle Motion Monitoring and Photothermal Treatment. Acs Nano.

[52] Wang C., Qu X., Zheng Q., Liu Y., Tan P., Shi B., Ouyang H., Chao S., Zou Y., Zhao C. (2021). Stretchable, Self-Healing, and Skin-Mounted Active Sensor for Multipoint Muscle Function Assessment. Acs Nano.

[53] Du S., Suo H., Xie G., Lyu Q., Mo M., Xie Z., Zhou N., Zhang L., Tao J., Zhu J. (2022). Self-powered and photothermal electronic skin patches for accelerating wound healing. Nano Energy.

[54] Deng L., Deng Y. (2023). A flexible triboelectric nanogenerator based on PVA/PTT/LiCl conductive hydrogel for gait monitoring in basketball. AIP Adv..

[55] Zhu K.-R., Wu L.-X., Liu M.N., Li C.L., Song W.-Z., Wei K.-Q., Zhang J., Ramakrishna S., Long Y.-Z. (2024). Triboelectric nanogenerator based on multi-component crosslinked network hydrogel for intelligent human motion sensing. Chem. Eng. J..

[56] Tian Z., Zhu Z., Yue S., Liu Y., Li Y., Yu Z.-Z., Yang D. (2024). Self-powered, self-healing, and anti-freezing triboelectric sensors for violation detection in sport events. Nano Energy.

[57] Guo X., Yang F., Sun X., Bai Y., Liu G., Liu W., Wang R., He X. (2022). Anti-Freezing Self-Adhesive Self-Healing Degradable Touch Panel with Ultra-Stretchable Performance Based on Transparent Triboelectric Nanogenerators. Adv. Funct. Mater..

[58] Wu W., Ren Y., Jiang T., Hou L., Zhou J., Jiang H. (2022). Anti-drying, transparent, ion-conducting, and tough organohydrogels for wearable multifunctional human-machine interfaces. Chem. Eng. J..

[59] Liu R., Liu Y., Fu S., Cheng Y., Jin K., Ma J., Wan Y., Tian Y. (2024). Humidity Adaptive Antifreeze Hydrogel Sensor for Intelligent Control and Human-Computer Interaction. Small.

[60] Zhu J., Zhu M., Shi Q., Wen F., Liu L., Dong B., Haroun A., Yang Y., Vachon P., Guo X. (2020). Progress in TENG technology-A journey from energy harvesting to nanoenergy and nanosystem. EcoMat.

[61] Wang Z.L., Wu W. (2012). Nanotechnology-Enabled Energy Harvesting for Self-Powered Micro-/Nanosystems. Angew. Chem. Int. Ed..

[62] Jiang D., Lian M., Xu M., Sun Q., Xu B.B., Thabet H.K., El-Bahy S.M., Ibrahim M.M., Huang M., Guo Z. (2023). Advances in triboelectric nanogenerator technology-applications in self-powered sensors, Internet of things, biomedicine, and blue energy. Adv. Compos. Hybrid Mater..

[63] Liu D., Huyan C., Wang Z., Guo Z., Zhang X., Torun H., Mulvihill D., Xu B.B., Chen F. (2023). Conductive polymer based hydrogels and their application in wearable sensors: A review. Mater. Horiz..

[64] Wu C., Wang A.C., Ding W., Guo H., Wang Z.L. (2019). Triboelectric Nanogenerator: A Foundation of the Energy for the New Era. Adv. Energy Mater..

[65] Cheng T., Shao J., Wang Z.L. (2023). Triboelectric nanogenerators. Nat. Rev. Methods Primers.

[66] Liu Z., Li H., Shi B., Fan Y., Wang Z.L., Li Z. (2019). Wearable and Implantable Triboelectric Nanogenerators. Adv. Funct. Mater..

[67] Choi Y.S., Kim S.-W., Kar-Narayan S. (2021). Materials-Related Strategies for Highly Efficient Triboelectric Energy Generators. Adv. Energy Mater..

[68] Chun J., Ye B.U., Lee J.W., Choi D., Kang C.-Y., Kim S.-W., Wang Z.L., Baik J.M. (2016). Boosted output performance of triboelectric nanogenerator via electric double layer effect. Nat. Commun..

[69] Mi H.-Y., Jing X., Wang Y., Shi X., Li H., Liu C., Shen C., Turng L.-S., Gong S. (2020). Poly (Butyl acrylate)-*co*-(butyl methacrylate) as Transparent Tribopositive Material for High-Performance Hydrogel-Based Triboelectric Nanogenerators. ACS Appl. Polym. Mater..

[70] Zhang Y., Xue C., Zhang Y., Zhang Q., Zhang K., Liu Y., Shan Z., Qiu W., Chen G., Li N. (2024). Cocktail effect of ionic patch driven by triboelectric nanogenerator for diabetic wound healing. Chin. Chem. Lett..

[71] Sheng F., Zhang B., Zhang Y., Li Y., Cheng R., Wei C., Ning C., Dong K., Wang Z.L. (2022). Ultrastretchable Organogel/Silicone Fiber-Helical Sensors for Self-Powered Implantable Ligament Strain Monitoring. ACS Nano.

[72] Zhang Z., Bai Y., Xu L., Zhao M., Shi M., Wang Z.L., Lu X. (2019). Triboelectric nanogenerators with simultaneous outputs in both single-electrode mode and freestanding-triboelectric-layer mode. Nano Energy.

[73] Niu S., Liu Y., Wang S., Lin L., Zhou Y.S., Hu Y., Wang Z.L. (2014). Theoretical Investigation and Structural Optimization of Single-Electrode Triboelectric Nanogenerators. Adv. Funct. Mater..

[74] Li Y., Cheng G., Lin Z.-H., Yang J., Lin L., Wang Z.L. (2015). Single-electrode-based rotationary triboelectric nanogenerator and its applications as self-powered contact area and eccentric angle sensors. Nano Energy.

[75] Zhang W., Wang P.-L., Ji X.-X., Huang L.-Z., Cao D.-Q., Li J., Ma M.-G. (2024). Ultrastretchable and adhesive MXene-based hydrogel for high-performance strain sensing and self-powered application. Compos. Part A Appl. Sci. Manuf..

[76] Di X., Hou J., Yang M., Wu G., Sun P. (2022). A bio-inspired, ultra-tough, high-sensitivity, and anti-swelling conductive hydrogel strain sensor for motion detection and information transmission. Mater. Horiz..

[77] Han X., Lu T., Zhang Z., Wang H., Lu S. (2023). Tremella polysaccharide-based conductive hydrogel with anti-freezing and self-healing ability for motion monitoring and intelligent interaction. Int. J. Biol. Macromol..

[78] Zhao L., Ling Q., Fan X., Gu H. (2023). Self-Healable, Adhesive, Anti-Drying, Freezing-Tolerant, and Transparent Conductive Organohydrogel as Flexible Strain Sensor, Triboelectric Nanogenerator, and Skin Barrier. ACS Appl. Mater. Interfaces.

[79] Zhang P., Zhao C., Zhao T., Liu M., Jiang L. (2019). Recent Advances in Bioinspired Gel Surfaces with Superwettability and Special Adhesion. Adv. Sci..

[80] Yu R., Li M., Li Z., Pan G., Liang Y., Guo B. (2022). Supramolecular Thermo-Contracting Adhesive Hydrogel with Self-Removability Simultaneously Enhancing Noninvasive Wound Closure and MRSA-Infected Wound Healing. Adv. Healthc. Mater..

[81] Han L., Lu X., Liu K., Wang K., Fang L., Weng L.-T., Zhang H., Tang Y., Ren F., Zhao C. (2017). Mussel-Inspired Adhesive and Tough Hydrogel Based on Nanoclay Confined Dopamine Polymerization. Acs Nano.

[82] Ling Q., Liu W., Liu J., Zhao L., Ren Z., Gu H. (2022). Highly Sensitive and Robust Polysaccharide-Based Composite Hydrogel Sensor Integrated with Underwater Repeatable Self-Adhesion and Rapid Self-Healing for Human Motion Detection. ACS Appl. Mater. Interfaces.

[83] Li J., Geng L., Wang G., Chu H., Wei H. (2017). Self-Healable Gels for Use in Wearable Devices. Chem. Mater..

[84] Ma Y., Liu K., Lao L., Li X., Zhang Z., Lu S., Li Y., Li Z. (2022). A stretchable, self-healing, okra polysaccharide-based hydrogel for fast-response and ultra-sensitive strain sensors. Int. J. Biol. Macromol..

[85] Long Y., Jiang B., Huang T., Liu Y., Niu J., Wang Z.L., Hu W. (2023). Super-Stretchable, Anti-Freezing, Anti-Drying Organogel Ionic Conductor for Multi-Mode Flexible Electronics. Adv. Funct. Mater..

[86] Sun H., Zhao Y., Wang C., Zhou K., Yan C., Zheng G., Huang J., Dai K., Liu C., Shen C. (2020). Ultra-Stretchable, durable and conductive hydrogel with hybrid double network as high performance strain sensor and stretchable triboelectric nanogenerator. Nano Energy.

[87] Zhang Y., He X., Xu C. (2023). A multifunctional hydrogel-based strain sensor and triboelectric nanogenerator for running monitoring and energy harvesting. APL Mater..

[88] Dong L., Wang M., Wu J., Zhu C., Shi J., Morikawa H. (2022). Stretchable, Adhesive, Self-Healable, and Conductive Hydrogel-Based Deformable Triboelectric Nanogenerator for Energy Harvesting and Human Motion Sensing. ACS Appl. Mater. Interfaces.

[89] Luo X., Zhu L., Wang Y.-C., Li J., Nie J., Wang Z.L. (2021). A Flexible Multifunctional Triboelectric Nanogenerator Based on MXene/PVA Hydrogel. Adv. Funct. Mater..

[90] Shao W., Zhang L., Jiang Z., Xu M., Chen Y., Li S., Liu C. (2022). Bioinspired conductive structural color hydrogels as a robotic knuckle rehabilitation electrical skin. Nanoscale Horiz..

[91] Li W., Wu S., Li S., Zhong X., Zhang X., Qiao H., Kang M., Chen J., Wang P., Tao L.-Q. (2023). Gesture Recognition System Using Reduced Graphene Oxide-Enhanced Hydrogel Strain Sensors for Rehabilitation Training. ACS Appl. Mater. Interfaces.

[92] Sun T.-C., Ning X.-C., Ramakrishna S., Long Y.-Z., Zhang J. (2023). Supratough and stretchable hydrogels with time-space controllability for athletic rehabilitation. Chem. Eng. J..

[93] Wang S., Zhang Y. (2022). A functional triboelectric nanogenerator based on the LiCl/PVA hydrogel for cheerleading training. Mater. Technol..

[94] Gai L., Wang F., Zhou F. (2022). A Stretchable Triboelectric Nanogenerator Integrated Ion Coagulation Electrode for Cheerleading Monitoring. J. Electron. Mater..

[95] Yang J., Wang H. (2023). A hydrogel triboelectric nanogenerator with self-healing function to obtain bio-mechanical energy and boxing training monitoring. J. Mater. Sci-Mater. Electron..

[96] Mao Y., Sun F., Zhu Y., Jia C., Zhao T., Huang C., Li C., Ba N., Che T., Chen S. (2022). Nanogenerator-Based Wireless Intelligent Motion Correction System for Storing Mechanical Energy of Human Motion. Sustainability.

[97] Sun F., Zhu Y., Jia C., Ouyang B., Zhao T., Li C., Ba N., Li X., Chen S., Che T. (2022). A Flexible Lightweight Triboelectric Nanogenerator for Protector and Scoring System in Taekwondo Competition Monitoring. Electronics.

[98] Yang Y., Zhao Y. (2024). A triboelectric nanogenerator based on flexible zwitterionic ionic conductive hydrogel for running training monitoring. Mater. Des..

[99] Zhu Y., Sun F., Jia C., Zhao T., Mao Y. (2022). A Stretchable and Self-Healing Hybrid Nano-Generator for Human Motion Monitoring. Nanomaterials.

[100] Zhou Z., Yuan W. (2023). Functionally integrated conductive organohydrogel sensor for wearable motion detection, triboelectric nanogenerator and non-contact sensing. Compos. Part A Appl. Sci. Manuf..

[101] Rahman M.T., Rahman M.S., Kumar H., Kim K., Kim S. (2023). Metal-Organic Framework Reinforced Highly Stretchable and Durable Conductive Hydrogel-Based Triboelectric Nanogenerator for Biomotion Sensing and Wearable Human-Machine Interfaces. Adv. Funct. Mater..

[102] Wu J., Teng X., Liu L., Cui H., Li X. (2024). Eutectogel-based self-powered wearable sensor for health monitoring in harsh environments. Nano Res..

[103] Lu Z., Jia C., Yang X., Zhu Y., Sun F., Zhao T., Zhang S., Mao Y. (2022). A Flexible TENG Based on Micro-Structure Film for Speed Skating Techniques Monitoring and Biomechanical Energy Harvesting. Nanomaterials.

[104] Lu Z., Xie Z., Zhu Y., Jia C., Zhang Y., Yang J., Zhou J., Sun F., Mao Y. (2022). A Stable and Durable Triboelectric Nanogenerator for Speed Skating Land Training Monitoring. Electronics.

[105] Yang Y., Sun H., Shi C., Liu Y., Zhu Y., Song Y. (2023). Self-healing hydrogel with multiple adhesion as sensors for winter sports. J. Colloid Interface Sci..

[106] Lei T., Wang Y., Zhang Q., Wang H., Duan X., Yan J., Xia Z., Wang R., Shou W., Li X. (2024). Ultra-stretchable and anti-freezing ionic conductive hydrogels as high performance strain sensors and flexible triboelectric nanogenerator in extreme environments. Nano Energy.

[107] Fu Z., Li D., Liu H., Liu R., Lyu Q., Han Y., Wang Y., Zhang K., Chen G., Tian Y. (2024). Antifreeze protein-based ultrastretchable and highly sensitive hydrogel for human-machine interaction. Chem. Eng. J..

[108] Xie Z., Dai Y., Wen Y., Zhang M., Tu M., Sun F., An Z., Zhao T., Liu B., Mao Y. (2024). Hydrogel-based flexible degradable triboelectric nanogenerators for human activity recognition. Sustain. Mater. Technol..

[109] Lu W.-X., Fang P., Zhu M.-L., Zhu Y.-R., Fan X.-J., Zhu T.-C., Zhou X., Wang F.-X., Chen T., Sun L.-N. (2023). Artificial Intelligence-Enabled Gesture-Language-Recognition Feedback System Using Strain-Sensor-Arrays-Based Smart Glove. Adv. Intell. Syst..

[110] Wang H., Ding Q., Luo Y., Wu Z., Yu J., Chen H., Zhou Y., Zhang H., Tao K., Chen X. (2024). High-Performance Hydrogel Sensors Enabled Multimodal and Accurate Human-Machine Interaction System for Active Rehabilitation. Adv. Mater..

[111] Goh G.L., Zhang H.N., Chong T.H., Yeong W.Y. (2021). 3D Printing of Multilayered and Multimaterial Electronics: A Review. Adv. Electron. Mater..

[112] Patil I.G., Thakur K., Nath S.S., Sundriyal P. (2024). 3D-printed energy harvesting devices for flexible and wearable electronics. Sustain. Energy Fuels.

